# Supplementary material for: Adapting spatiotemporal gait symmetry to functional electrical stimulation during treadmill walking
Source: PLoS One. 2024 Oct 18;19(10):e0312285. doi: 10.1371/journal.pone.0312285 (PMC11488702; doi:10.1371/journal.pone.0312285)
Supplement: Attachment — Submitted filename: Response to Reviewers Comments.docx [file pone.0312285.s002.docx]

**Response to Reviewer's Comments**
===============================
We would like to thank the reviewers for their valuable comments. The reviewers’ comments are listed below in black, and the authors’ response is blue.

**Reviewer #1:** Reviewer #2: Thanks for taking on the feedback positively and for adapting the manuscript!

Some small things - I noticed that the abstract in the manuscript submission system is not adapted to reflect the changes made.

>> Thank you for the information. Honestly, we're unsure why the changes we made didn't reflect in the revised PDF. We hope that this time, the submission system will correctly update the abstract with the revisions.

There is also still some incongruency between the first paragraph of the discussion and the abstract, in regard to whether the perturbation induces gait symmetry or asymmetry adaptations. Apart from these textual edits, I have no concerns that should keep this from being published.

>> We aimed to induce gait asymmetry during training through FES perturbation, ultimately facilitating the adaptation of asymmetric gait patterns. We have revised the first sentence of the Discussion, which now reads:

*“In this study, we proposed a novel approach to utilizing FES as a perturbation method for altering symmetric gait patterns and evaluated the potential benefits of the FES perturbation by examining the extent of spatiotemporal gait asymmetry adaptation during treadmill walking.”*

Congratulations and all the best!

>> Thank you for taking the time to review our manuscript. We greatly appreciate your feedback, which has helped us make significant improvements.
